# Trauma-Informed Care on mental health wards: staff and service user perspectives

**DOI:** 10.3389/fpsyg.2025.1578821

**Published:** 2025-09-19

**Authors:** Faye Nikopaschos, Orla Gibbons, Emma Bailey, Anna Foxall, Camilla Giachero, Gail Burrell

**Affiliations:** Harrow Adult Mental Health, Central and North West London NHS Foundation Trust (CNWL), London, United Kingdom

**Keywords:** Trauma-Informed Care (TIC), Power Threat Meaning Framework (PTMF), trauma, stabilisation, Team Formulation, inpatient

## Abstract

**Aim:**

This is the second study from a two-part evaluation into the impact of introducing Trauma-Informed Care (TIC) to a National Health Service (NHS) adult acute inpatient setting. The project consisted of two linked practices: Power Threat Meaning Framework (PTMF) Team Formulation, and a trauma-informed training program for staff, combined with the provision of Psychological Stabilisation resources. The first paper reported significant reductions in self-harm incidents and the use of restrictive practice on the wards. This paper aimed to elucidate experiences and mechanisms of change through a qualitative exploration of the impact of these new practices on staff and service users.

**Method:**

Staff (*N* = 7) and service users (*N* = 8) took part in semi-structured interviews about their experiences of the TIC project, which were subjected to Thematic Analysis.

**Results:**

Thematic analysis of staff interviews (*N* = 7) identified three main themes: ‘Changes in Knowledge and Understanding’, ‘Changes in Engagement and Practice’ and ‘Barriers and Constraints’. Thematic analysis of the service user interviews identified two main themes: ‘The Benefits of Stabilisation Interventions’ and ‘Trauma-Informed Care Helped my Recovery.’

**Conclusion:**

Staff felt that the trauma-informed practices provided a meaningful conceptual framework for the better understanding of service users’ difficulties, which led to increased compassion and a wider range of helpful responses toward service user distress. Service users reported that they had gained new insights and skills, and been helped by their admission. The findings are considered in relation to the wider literature on TIC, and the benefits and challenges of introducing culture change in inpatient settings.

## 1 Introduction

### 1.1 Trauma-Informed Care

Trauma-Informed Care (TIC) is an emerging paradigm within mental health services that recognizes the pervasiveness and lasting impact of trauma on a large majority of mental health service users (Sweeney et al., 2018). TIC is grounded in four core components, often referred to as the “Four R’s.” ‘Realizing’ the widespread impact of trauma and understanding paths for recovery; ‘recognizing’ the signs and symptoms of trauma in service users, staff, and others involved; ‘responding’ by integrating this knowledge into practice and policy; and ‘resisting’ re-traumatization by actively avoiding practices that may retraumatize individuals. These components are underpinned by several key principles—ensuring safety, building trustworthiness, supporting choice, fostering collaboration, promoting empowerment, and addressing cultural, historical, and gender considerations—which together aim to create inclusive, responsive environments that support healing and engagement (Office for Health Improvement and Disparities, 2022).

Within a trauma-informed perspective, ‘symptoms’ are understood as attempts to cope within the context of adverse life experiences (Sweeney et al., 2016). The approach is supported by an evidence-base demonstrating the relationship between maltreatment experienced in childhood and a range of subsequent mental health and other difficulties (e.g., Felitti et al., 1998; Kessler et al., 2010; Mauritz et al., 2013; Heffler et al., 2020). This has led to the development of practices that promote recovery and are less likely to be re-traumatizing (Harris and Fallot, 2001).

In mental health settings, service users who have experienced sexual or physical abuse in childhood are typically prescribed higher rates of medication, have longer and more frequent hospital admissions, and are more likely to self-harm and attempt suicide (Anda et al., 2007; Gibb et al., 2003; Kessler et al., 2010). Other forms of adversity such as material poverty, social disadvantage and discrimination are also associated with an increased likelihood of contact with mental health services (Pickett and Wilkinson, 2010).

The Long Term Plan (NHS, 2019) recommends the implementation of TIC in mental health services. However, longstanding challenges remain, including the availability of comprehensive staff training on TIC; prioritization of trauma awareness within institutional cultures; and difficulty translating TIC principles into concrete practices (Muskett, 2014).

In terms of research, some studies suggest that the beneficial effects of TIC include reduction in post-traumatic stress, increased coping skills, shorter inpatient stays and less use of restrictive practices by staff (e.g., Azeem et al., 2011; Han et al., 2021; Messina et al., 2014; Muskett, 2014). So far, only one study has explored changes in staff members’ understandings of the nature of distress as a result of transition to TIC (Chandler, 2008). Similarly to our first study (Nikopaschos et al., 2023) it found reductions in restraint and seclusion, and attributed these in part to the staff’s ‘changed perspectives’ (p.366), as the current study also suggests. It is noted that the current evidence-base on TIC is largely limited to a small number of quasi-experimental studies within the United States (US) with varying clinical settings, trauma-informed strategies and outcome measures (Goldstein et al., 2024). While there is growing work internationally, including in Australia (Richards et al., 2019), the overall evidence remains heterogeneous and in need of further robust evaluation.

TIC has been criticized for its conceptual ambiguity, which complicates efforts to define its core components and evaluate its implementation (Bargeman et al., 2022). Rather than offering a clearly delineated framework, TIC is often described in broad, aspirational terms that resist operationalization. Sweeney et al. (2018) argue that becoming trauma-informed does not necessarily entail specific, measurable actions but instead reflects a ‘shift in ideology’—a fundamental change in how practitioners understand, relate to, and work with service users (p. 330). While this emphasis on values and cultural transformation can be empowering, critics contend that the absence of a consistent definition risks diluting its impact and allows for superficial or symbolic adoption without meaningful systemic change (Hopper et al., 2010; Watson, 2017). This conceptual looseness also raises concerns about accountability and fidelity in service delivery, prompting calls for clearer theoretical foundations and practice-based standards (Wilcox et al., 2021).

### 1.2 The Power Threat Meaning Framework

The Power Threat Meaning Framework (PTMF) was published by the British Psychological Society in 2018 (Johnstone et al., 2018). This Framework outlines four key processes in the development of what are termed mental health difficulties and experiences of distress more generally: the negative operation of power, the forms of threat this poses, the meanings that are attributed to these events and circumstances, and the ways that people may respond in order to survive and protect themselves. Mental health ‘symptoms’ are therefore understood as patterns of meaning-based threat responses, and as strategies for surviving adversity. This forms the basis for construction, or co-construction, of a personal story, narrative or formulation. The PTMF is compatible with TIC, but goes further than some versions of this approach in that it poses a direct challenge to the dominant medical paradigm of psychological and emotional distress (Johnstone et al., 2018). Since it is a relatively recent publication and offers a set of principles rather than specific guidance for practice, there is little research on its clinical applications to date (Johnstone et al., 2019; Gallagher et al., 2024).

### 1.3 The project

This paper is the second part of a larger evaluation of a project that introduced TIC into a National Health Service (NHS) adult acute inpatient mental health unit in North London. The first study reported quantitative data (Nikopaschos et al., 2023) and this one presents qualitative data.

Both studies took place within the above setting, consisting of two adult acute mental health wards. The ward teams comprise Occupational Therapists, Psychiatrists, Nurses, Health Care Assistants, Peer Support Workers and Clinical Psychologists. Since July 2018, the service has been undergoing transition towards a model of TIC, now in its sixth year of implementation. Drivers for the changes on the wards included an overemphasis on restrictive practice and dispensing medication as a first response to distress, with limited access to psychological frameworks for understanding service users’ distress or to psychosocial interventions to support recovery. Wider influences included the national policy drive for the implementation of services using TIC (NHS, 2019). In response, the service introduced a weekly team formulation meeting, informed by the PTMF, and a corresponding weekly training program for staff on the effects of trauma and the use of stabilisation strategies to support service users with their distress. This was supported by accessible trauma-informed educational resources (Trauma-Informed Stabilisation Manual, Nikopaschos et al., 2020), which were offered to all service users. For a detailed overview of the project, see Nikopaschos et al. (2023). The two main trauma-informed practices are described below.

#### 1.3.1 PTMF Team Formulation

From July 2018, team formulation meetings have taken place for an hour once a week, alternating between the two wards. All multi-disciplinary team members attend, and new service users are discussed each week as part of developing formulation-based care plans. The meetings are facilitated by senior staff members from the multi-disciplinary team (MDT) including the Principal Psychologist, the Borough Director who is an Occupational Therapist, the Matron and the Lead Occupational Therapist. Meetings are informed by the PTMF core questions (Appendix A) and structured in accordance with a standard protocol (Appendix B) and Quality Measure (available from the authors), which were drawn up to ensure consistency and attendance to all key areas. The team discussion is intended to help make sense of difficulties such as suicidality, hearing voices, anger, panic and dysregulation as threat responses to adverse life experiences.

#### 1.3.2 Trauma-Informed training

In addition to the team formulation meetings, a rolling 12-week staff training program was introduced weekly from November 2018, informed by the Three Stage Model of trauma recovery (Herman, 2015). The three stages are: (1) the establishment of safety and coping strategies, sometimes known as Stabilisation; (2) active processing of trauma memories; and (3) rebuilding relationships and everyday life. Since Stabilisation is the most urgent need for services users on adult acute inpatient wards, all staff were trained in 10 safety-based skills to support them to manage service users’ distress, along with a general introduction to TIC and the PTMF. The skills comprised Self-Compassion, Soothing and Safety, Mindfulness, Effective Communication, Breathing and Relaxation, Food and Sleep, Distraction and Distancing, Valued Activity, Grounding and Maintaining Wellbeing. A Stabilisation Manual, which covered these 10 skills alongside information on the trauma-informed model, was also developed (Nikopaschos et al., 2020) and delivered to service users by inpatient staff through both individual and group sessions. Staff also used these skills to de-escalate high distress incidents on the ward and all service users had access to the manual to review in their own time.

#### 1.3.3 Project outcomes

The main outcomes after 4 years, as reported by Nikopaschos et al., 2023, were as follows: significant reductions in the monthly numbers of incidents of self-harm (*p* < 0.01; *r* = 0.42), seclusion (*p* < 0.05; *r* = 0.30) and restraint (*p* < 0.05; *d* = 0.55) on the two inpatient wards. A number of mechanisms of change were hypothesized. The present study is a qualitative analysis of staff and service users’ experiences of the project, which aimed to explore and elucidate the change processes reported in the first study.

## 2 Materials and methods

### 2.1 Design

The study employed a qualitative methodology and was conducted in two parts. In the first, data were collected using individual semi-structured interviews with seven staff members. In the second, data were collected using individual semi-structured interviews with eight service users. The two sets of interviews used different questions and prompts, as appropriate for the participant groups, but in both cases, Thematic Analysis was employed to draw out the themes. Procedures and findings from the two groups – staff and service users – are reported separately in this section.

The project was registered with the NHS Trust’s (Central and North West London NHS Foundation Trust [CNWL]) Information and Governance Team. A Data Impact Assessment (DIPA) was completed and approved by this team.

### 2.2 Evaluation aims

Several possible mechanisms of change were suggested by Nikopaschos et al. (2023) to account for the main outcomes (significant reductions in self-harm and restrictive interventions) reported in the original study. In line with previous findings about team formulation, it was suggested that the team formulations may have helped the team to:

- better understand service users from a trauma-informed perspective
- develop novel ways of managing distress on the ward
- increase their understanding of the potential for re-traumatization, by making explicit service users’ past experiences of trauma and their meaningful links to current behavior
- increase staff understanding of the importance for creative alternatives to restrictive intervention
- strengthen relationships between staff and service users
- increase levels of empathy toward service users
- improve staff teamwork and communication.

In relation to the introduction to TIC and stabilisation training, it was hypothesized that these factors may have helped to bring about reductions in self-harm, seclusion and restraint by supporting:

- reduced overall levels of service user distress
- service users to feel more able to cope with high levels of distress
- staff to respond to and de-escalate distress more effectively.

The current study aimed to explore staff and service user experiences of and perspectives on the project, and in doing so, elucidate the possible mechanisms of change for the outcomes reported in the first study. It was hoped that this might:

- contribute to understanding experiences and mechanisms of change in relation to introducing TIC in inpatient settings
- add to the current evidence-base about the clinical applications of the PTMF.

### 2.3 Epistemological position

This study adopted a Critical Realist approach to epistemology, which provides a position between positivist and constructionist thinking (Fletcher, 2017; Willis, 2023). Applied here, the approach treats participants’ descriptions as valid depictions of their understanding and practice, while acknowledging that this depiction does not necessarily constitute a direct reflection of the phenomenon under investigation (Willis, 2023).

### 2.4 Reflexivity statement

The research team comprised clinical psychologists and an occupational therapist, who value psychological interventions and regard the TIC model as a meaningful framework for understanding psychological distress. We acknowledge that these professional backgrounds and theoretical orientations may have influenced the research process, including data collection, coding and interpretation.

To manage potential bias, the team engaged in ongoing reflexive practice throughout the study. This included regular discussions to critically examine how our perspectives and assumptions might shape the analysis. In addition, we employed peer debriefing within the research team, where emerging codes and themes were reviewed collaboratively to challenge individual biases and promote analytical rigor.

The study adopted a Critical Realist epistemological stance, which acknowledges an objective reality shaped by social contexts and individual experiences. This stance informed our thematic analysis by encouraging us to explore not only participants’ reported experiences but also underlying mechanisms and structures that influence those experiences. It allowed us to interpret themes as reflections of real phenomena while recognizing the partial and situated nature of knowledge.

### 2.5 Staff interviews: Recruitment and description of sample

In total, 27 members of staff attended the first rotation of rolling 12-week training programs, which took place from November 2018. An average of five staff members attended each training session. Attendance fluctuated across the 12 weeks, with no professional group or individual attending every session. Staff members who had attended more than three training and five team formulation sessions were invited to be interviewed, to ensure sufficient experience of team formulation and TIC. Of the 27 who attended the training, seven met these criteria and were invited to take part. The participants had attended an average of 14 formulation sessions (range: 6–30) and five training sessions (range: 3–8) (Table 1).

**Table 1:** Participant characteristics.

*n*	Gender	Profession
7	M = 1 F = 6	OT = 2 Nurse = 3 HCA = 2

### 2.6 Service user interviews: Recruitment and description of sample

Service users who had had been discharged within 6 months of the start of data collection were invited, to ensure that they had received the trauma-informed model of care. They were also required to have attended at least five stabilisation sessions, either group or individual or a mixture of both, while in hospital. To protect vulnerable participants, anyone who appeared to be experiencing a current mental health crisis was excluded. This was checked by looking at patient record systems before contacting service users for feedback. Fifteen service users met inclusion criteria and were contacted; of these, five did not answer the calls and two declined to participate. See Table 2 for participants’ demographics.

**Table 2:** Participant demographics.

*n*	Gender	Ethnicity	Age (years)
8	M = 5 F = 3	Asian = 4 Black = 1 White = 3	Mean = 42.5 Min = 29 Max = 52

### 2.7 Data collection

Semi-structured interview schedules (Appendices C, D) were developed for each group of participants. An expert by experience provided consultation to the service user evaluation and reviewed the interview schedule. Feedback was given on the language and wording of questions and the topics covered, to ensure interview questions were accessible to service users. OG and AF carried out the staff interviews and EB and CG carried out the service user interviews. Contact numbers were made available to service users in the event of the interview causing distress.

The main areas addressed in the staff member interviews were:

- How the team formulation and TIC training sessions had changed their understanding of the development of mental health difficulties.
- How the team formulation and TIC training sessions had influenced their perspectives on their practice and engagement with service users.

Individual interviews with staff took place face to face over a period of 2 weeks, 1 month after the completion of the first 12-week training cycle and lasted between 15 and 30 min.

The main areas addressed in the service user interviews were:

- What they had found helpful or unhelpful about using stabilisation interventions.
- How they had experienced TIC during their admission.

Interviews took place over the telephone and lasted between 10 and 20 min.

### 2.8 Data analysis

For both sets of interviews, the qualitative data were analyzed using Thematic Analysis employing Braun and Clarke’s (2006) six-phase guidelines. Thematic Analysis is well suited to exploring how participants conceptualize an area of practice and how they make sense of its associated interventions (Braun and Clarke, 2006). Initial codes were derived by transcribing the data, reading through several times, making connections, and noticing patterns. This involved a recursive process, moving between a data and theory-driven approach (Braun and Clarke, 2006). The resulting codes with accompanying extracts were collated (examples from the staff interviews in Appendix E). Initial themes, sub-themes and accompanying extracts from the service user interviews can be found in Appendix F. Grouping and comparing codes generated initial themes and sub-themes, which were reviewed and refined to provide a coherent narrative to the data. OG and AF performed the analysis of the staff interviews and EB and CG performed the analysis of the service user interviews. Both sets of themes were validated by a psychologist external to the project, in line with Braun and Clarke's (2006) recommendation for Thematic Analysis.

## 3 Results

### 3.1 Staff interviews

Three main themes were identified with corresponding sub-themes. Each theme is described below, with exemplar extracts in accordance with Braun and Clarke’s (2006) guidelines. Repeated words and hesitations have been removed from the extracts, for readability. The use of an ellipsis indicates that words have been removed (Table 3).

**Table 3:** Staff interviews: Themes and sub-themes.

Theme	Sub-themes
Changes in knowledge and understanding	A new framework linking trauma and distress
	Distress as a threat response
	Limitations of a narrowly diagnostic approach
Changes in engagement and practice	Increased compassion
	Establishing safety
	De-escalating distress
	Talking about the past
Barriers and constraints	Demands of the ward environment
	Need for a whole service approach

#### 3.1.1 Theme one: Changes in knowledge and understanding

##### 3.1.1.1 Sub-theme: A new framework linking trauma and distress

Participants described how PTMF Team Formulation and TIC training had provided new and compelling information and perspectives, serving to deepen their understanding of the relationship between past trauma and mental health problems. Although these ideas were not completely new to participants, they felt they now had a more comprehensive insight into the pervasiveness and lasting damage of traumatization. Participants valued being better able to help service users connect their early experiences of mistreatment with the development of later relational difficulties and patterns of response. Overall, they described a shift in focus from what is happening now, to a curiosity about what had happened prior to the admission and in the service user’s life overall. This was a contrast to these issues being obscured behind a diagnosis.

*I was very shocked by the percentages [on prevalence rates] ... it's made me think that a lot of people in mental health services have been abused ... it was really eye opening.* (P2)

*You hear all these patients talk about their traumas, but I didn't realize that was kind of connected. [Following the Team Formulation and training] I just realized that a lot of these patients have a lot in common, I think the majority of their issues are due to childhood traumas.* (P5)

*Before attending, yeah we know that people are depressed because of a [social] issue but doesn’t really give you the full understanding.* (P2)

*You focus on what they're feeling rather than on symptoms and diagnosis … The [PTMF] framework helps to understand them as individuals and address what's behind the diagnosis.* (P1)

##### 3.1.1.2 Sub-theme: Distress as a threat response

As a result of this new conceptualization, participants now saw service users’ reactions and behavior very differently. Staff described how the team formulation meetings transformed the team’s understandings, by framing service user reactions not as ‘symptoms’ but as intelligible attempts to survive difficult situations. The concept of threat responses was seen as very helpful in this respect.

*When we're discussing it as a team, people will come forward and say, ‘oh actually, they've had this happen to them before, and this is how they've learnt to respond to that’, everyone starts to think, ‘oh yeah, actually that makes sense and that fits as a threat response’.* (P4)

*[TIC] gives you so much more background to things that you would look at as symptoms ... you might look at something as challenging behavior, but when you actually look at what they responded to, to behave that way, and then tie it in with all their experiences throughout their life, it makes so much more sense.* (P4)

*You get to understand why someone has reacted to you in a certain way … let's say it's bad behavior … it’s because of the trauma they've experienced, it's not me. It sort of makes you a little bit calmer, more understanding.* (P6)

*Understanding where this aggression or intimidation comes from … it comes from a place of fear, so through trauma – ‘I'll get you before you get me’ … being able to say to that person …‘you know this happened in the past, so it's quite understandable that you react like this’.* (P6)

##### 3.1.1.3 Sub-theme: Limitations of a narrowly diagnostic approach

Several participants reflected on the limitations of relying on a narrowly diagnostic approach, including not exploring the service user’s story, seeing behavior simply in terms of illness or symptomatology, and overemphasizing medication as a first response to distress. Many participants became aware of the potential for re-traumatization within the ward environment, for example by recreating a sense of fear or powerlessness. The concept of power imbalances within the PTMF team formulation meetings appeared to have been helpful in enabling participants to identify and reflect on the impact of power differentials on the ward.

*When an individual comes in for admission, we forget the whole history, we go straight to what they're presenting with … why not the history as well? You might be able to link it to why they've had the break down [resulting in admission].* (P2)

*We’re doing what’s been done to them for how many years … you’re just sort of reinforcing abuse that happens to them.* (P4)

*We're very focused on using medication, but it's not only medication that solves the problem.* (P2)

#### 3.1.2 Theme two: Changes in engagement and practice

##### 3.1.2.1 Sub-theme: Increased compassion

Participants reported that a trauma-informed perspective resulted in different emotional responses to patients, primarily increased feelings of compassion. Better understanding of trauma histories was described as leading to a greater ability to empathize with the unique challenges that trauma survivors face, and participants described the powerful transformation in staff members’ emotional responses and understandings that can take place during the team formulation sessions.

*You feel more compassionate … because you know exactly what went on, and you just think how you would feel, or a member of your family.* (P7)

*We'll start by talking about how we feel about this person, and then we'll go through the [PTMF] framework, then we'll talk at the end again [about] how we feel about that person, and everything's changed … people start by being like, ‘it's frustrating’, or ‘I've been intimidated by this person’, then at the end say ‘I feel sad, or compassionate’.* (P4)

*It made you aware that the background of people has a huge impact on how they're behaving … it helps with your compassion and your understanding as to why they are like that or why they are acting that way.* (P3)

##### 3.1.2.2 Sub-theme: Establishing safety

Participants demonstrated an awareness that a feeling of lack of safety is the core of the experience of trauma, and can persist beyond the traumatic experiences themselves. The majority of participants described making active use of the strategies introduced in the training to help service users gain a sense of safety and security. Stabilisation strategies were seen as highly beneficial in helping patients to access inner resources, and soothe and regulate themselves, during their admission. The ward environment and time spent with service users presented opportunities for using the strategies in action. The staff described creative ways of adapting the strategies for people’s specific needs. The emphasis here was on proactive, preventative strategies that were embedded into daily care routines and adapted to individual needs. Staff described using personalized stabilisation techniques—such as mindfulness, sensory items, and visualizations—not just during crises but as part of a wider effort to strengthen service users’ internal coping resources and sense of calm.

*We discussed using photos and stuff that remind them of a safe spot. We have a few patients with soothing boxes, which also works well, and most of them have stress balls.* (P3)

*I bring in mindfulness exercises … one of the patients was really upset … so I got one of the bubble blowers, I started to blow it and then she started to blow it as well, and that made her more relaxed … and kind of just focus [ed] on the bubbles.* (P5)

*He wanted to have something implanted in his mind to delete memories … what he wanted was to not focus on those negative things … he managed to identify which distancing and distraction techniques he could use, and yeah it worked.* (P1)

##### 3.1.2.3 Sub-theme: De-escalating distress

This theme focuses on how staff responded during episodes of heightened emotional distress or behavioral escalation. The trauma-informed approach shifted how staff understood and responded to these incidents—moving away from reactive or punitive responses, and toward a stance of curiosity, empathy and emotional regulation. Rather than taking it personally, or becoming emotionally aroused themselves, they felt they could stay calmer. They were now more likely to try and understand the emotions that the service user was struggling to manage, rather than resorting to medication. The stabilisation strategies also meant that staff had a range of ways of responding to service users’ distress. The staff described working in collaboration with the service user to identify what might be most helpful to them in a crisis. Unlike the ongoing work of establishing safety, de-escalation was described as responsive and dynamic, focused on managing crises in the moment.

*I got … more confidence to use those techniques … when someone is very distressed.* (P1)

*If I know that … they've done grounding techniques, or distraction and distancing, and I know which one's been really effective for them, then I can encourage them to look at them when they're really distressed and it might completely reverse that.* (P4)

*Because what we're not doing before was ‘How do you think we can help you? What makes you safe in the past?’, just little things like that.* (P2)

*Try to understand … ask ‘what’s happening, why do you feel this way?’ … There's this patient that gets distressed quite easily ... and becomes a bit aggressive, every time he was getting like that, I was reminding him – ‘remember your self-soothing items’.* (P1)

*If they were like showing a lot of anxiety or anger, you kind of learnt to look at it from different perspectives and that helped me come up with different interventions.* (P1)

##### 3.1.2.4 Sub-theme: Talking about the past

Several participants reported having felt anxious about how to talk to service users about their past. Staff described the usefulness of moving away from a stance of ‘What is wrong with you?’ to ‘What has happened to you?’ as a way to frame conversations. They were now more confident about doing so safely and appropriately, such as through active listening, allowing information to emerge gradually, and making links with past events. They were also offering more opportunities for service users to talk about their past experiences.

*The training helped a lot to understand what trauma is and how to talk about it with someone. You know this famous phrase, do not ask ‘What's wrong with you?’ but ‘What happened?’ It helped a lot to know how to approach that, because obviously talking about a person's trauma, it can be difficult.* (P1)

*I didn't really know what kind of questions were appropriate … I don't want to come across as judgmental … I just listen to what they have to say now, and then they slowly start opening up and telling me about their childhood.* (P5)

*I’ll make sure I’m asking more about what their childhood was like, what their upbringing was like and how they feel about it now.* (P4)

*It's like insight, giving someone [the understanding that] – ‘well, I know why I react to criticism really badly … because I was criticized when I was younger’ … It's looking at what's happened to you, that's how I would describe it to a patient.* (P6)

#### 3.1.3 Theme three: Barriers and constraints

##### 3.1.3.1 Sub-theme: Demands of the ward environment

The ward environment was frequently described as limiting the opportunity to use the stabilisation interventions, due to the intense and demanding nature of the work, the need to prioritize other targets, and pressure to discharge service users.

*Not enough hours in the day, not enough time … you have to prioritize KPIs [key performance indicators] … You do get fired up about how you want to do this, [but] sometimes you get grind (sic) down by the ward.* (P6)

*Another kind of barrier is the setting itself. Acute - everything can happen so fast. So you may come up with a plan, an amazing plan, like 'Today we're going to this, next week that’ ... and then they are discharged* (P1).

*It's finding the time to use the workbooks … it depends on what shift you're on … we've got ward round, the wards usually busy, it depends if you've got enough staff.* (P5)

##### 3.1.3.2 Sub-theme: Need for a whole service approach

Several participants reflected on the potential value of TIC as a unifying model for all ward staff, regardless of discipline. However, some felt the model was not yet fully integrated into the ward teams. They reported a disparity across staff members’ engagement and adoption of the model, and were concerned that this might limit the effectiveness of the strategies and the approach.

*Any staff can use it, it's not OT [Occupational Therapy] related, nursing related … it makes the whole team have the same language, the same approach.* (P1)

*It's not become the culture necessarily just yet … It's about all of us seeing [that] it’s our jobs to be doing these things … I can make a self-soothe box, but if the [other] staff don't know about it, they're not going to say to them – ‘What's in your self-soothe box?’* (P4)

### 3.2 Service user interviews

Two main themes were identified with corresponding sub-themes. The themes are described below, with exemplar extracts in accordance with Braun and Clarke’s (2006) guidelines. Repeated words and hesitations have been removed from the extracts, for readability. The use of an ellipsis indicates that words have been removed (Table 4).

**Table 4:** Service user interviews: Themes and sub-themes.

Theme	Sub-themes
Benefits of stabilisation interventions	New knowledge and skills to manage distress
	The manual is a helpful resource
	Individual versus group sessions
	Using stabilisation skills post-discharge
Trauma-Informed Care helped my recovery	New understandings of distress
	The importance of talking about difficult issues
	The staff were supportive
	The admission was much more helpful

#### 3.2.1 Theme one: The benefits of stabilisation interventions

##### 3.2.1.1 Subtheme: New knowledge and skills to manage distress

Participants described how the information and skills in the stabilisation manual helped them to manage their distress better. A range of skills were mentioned, with breathing and mindfulness being named most frequently.

*[The stabilisation interventions] helped me to understand what I was going through and how to manage it in case it comes back.* (P6)

*Meditation and mindfulness skills very helpful. I would get into a bit of a rage, get upset, then use the skill and it would calm me down.* (P3)

*I used them mainly when I had the sessions and in the evening time … I would use the techniques that I learnt then to keep me calm.* (P8)

*Very helpful. Because it’s not something that I knew. The skills, they teach you how to manage your stress, how to put it across to the doctors.* (P4)

*Self-compassion skill–had never heard of the term before. I don’t like myself very much, but we are who we are, and it helped me to deal with it.* (P3)

##### 3.2.1.2 Subtheme: The manual is a helpful resource

Most participants found the stabilisation manual a helpful and accessible resource, although some had not been able to make full use of it due to their mental state.

*Still open the manual up and read the skills, I have also spoken to other people about the skills and shown them … they also say they find it helpful.* (P6)

*The stabilisation interventions kept me going in hospital. The manual gave me something to work toward, I was able to achieve something.* (P1)

*I sort of read through [the manual] but didn’t really take a lot in … I would rush through it to say that I’ve done it … it’s my memory and that is so bad.* (P7)

##### 3.2.1.3 Subtheme: Individual versus group sessions

Some participants valued the group sessions as an opportunity to learn from each other. However, most participants found individual sessions an easier way of sharing their experiences.

*I was enjoying the groups, I was able to participate and talk about the issues and subjects that were covered.* (P1)

*I don’t really like talking about things in front of big groups of people. More comfortable in 1: 1s.* (P3)

##### 3.2.1.4 Subtheme: Using stabilisation skills post-discharge

Some participants reported difficulties continuing to use the stabilisation skills after discharge, as home settings were less structured. However, others found the home environment easier, and had continued to use the skills.

*Yeah, mainly because it’s the only thing I brought out the hospital with me, how to manage myself … I’ve got my plan on the wall at home, managing distress in the moment plan. Breathing, and just putting things in order, having a routine.* (P8)

*When you’re on the ward, it’s there, you know the groups are coming up, the 1: 1 sessions are in the afternoon, there is the reminder that it is going on. … Not in the forefront of my mind [since discharge], it’s not like I don’t want to be doing it because it doesn’t help, I just can’t remember it.* (P7)

#### 3.2.2 Theme 2: Trauma-Informed Care helped my recovery

##### 3.2.2.1 Subtheme: New understandings of distress

Some participants described a new way of understanding their mental health difficulties as arising from unresolved events, consistent with the trauma-informed perspective.

*We did my timeline (an exercise listing important past events), linking the past to my present, it really helped.* (P1)

*Yes, my history was taken into account by the team. It helped me to come to terms with the death of my father. Staff asked me about it, asked the right questions … I kept it in, a lot of guilt about the death of my father … Hospital and talking helped me to come through the other side.* (P3)

##### 3.2.2.2 Subtheme: The importance of talking about difficult issues

A number of participants said how helpful it was to talk through past events and traumas, although two service users felt that this was less helpful and focused on too much by staff.

*It made me feel a lot better because I’ve never really opened up to people, always bottled everything up. It was a good decision, I needed to open up to someone otherwise it would have made me worse.* (P8)

*It was good that someone was interested. I was overwhelmed doing the timeline … I was bottling it all up. That’s why I was slow and had my illness, because I don’t talk.* (P1)

*It’s okay for past to be taken into account. But keep on bringing up, numerous times. That is dragging person back in that mood or mind space where they are depressed.* (P4)

*The doctors asked me but I don’t want to think about the past … wanting to focus more positively about the future.* (P5)

##### 3.2.2.3 Subtheme: The staff were supportive

Several participants commented that the staff took a personal interest in them and their difficulties, and were encouraging and supportive.

*I can’t fault the time I had in there … They want to get to know you and get to know about your problems because then you know that’s when you can start to help people, rather than just reading from a manual, and box ticking … I felt like an individual not a tick on a sheet.* (P7)

*I had a bad experience when I was younger and I mentioned that to [staff member] and she took that into consideration and tried to sort me out … The nurses were also supportive. It was helpful.* (P8)

*Everyone was understanding, all the staff great. They all helped me in different ways. They were all great and all helped me with my recovery.* (P1)

##### 3.2.2.4 Subtheme: This admission was much more helpful

Some of the service users had experience of previous admissions. They felt TIC had made their recent admission much more helpful. Participants mainly commented on how this admission differed in that they learnt skills to manage their distress and were provided with opportunities to talk to staff.

*15 years ago. In the past when I was sectioned, it wasn’t really helpful. They didn’t go through skills. They did not talk about stabilisation like on this ward. This ward [was] very helpful.* (P4)

*There was a difference between hospitals. [X] hospital … it wasn’t really about talking, more about keeping busy. It’s a positive difference, the fact that I was able to talk during my admission. Talking helps.* (P1)

*Another one two years ago … I did not bother to engage. There was no staff around to talk to. This time I engaged and spoke to staff … I’m happy I did as it helped me.* (P3)

## 4 Discussion

The present study builds on an earlier quantitative evaluation, looking at the impact of introducing Trauma-Informed Care (TIC) in an NHS adult acute inpatient mental health unit in North London. As well as introducing staff to the rationale and principles of a trauma-informed approach, the project included team formulation meetings based on the Power Threat Meaning Framework, and the use of stabilisation resources. The quantitative evaluation (Nikopaschos et al., 2023) found significant reductions in seclusion, restraint and self-harm on the wards, while the current study aimed to explore staff and service user experiences of TIC and elucidate possible mechanisms of change, using individual semi-structured interviews.

The two sets of data presented in this study necessarily reflect different exposure to TIC. While both staff and service users learned about the trauma-informed model and stabilisation skills, the former had more in-depth training in the approach, along with the experience of attending team formulation meetings, and were reporting as members of the ward team. The latter group were recipients of the new approach. In addition, the service user interviews were much briefer and yielded less detailed information. However, the two sets of themes are complementary.

### 4.1 Themes: Summary of staff views

- The ward teams had been working within an approach described by staff as mainly reliant on diagnosis and medication, which as a result, prevented people’s stories and contexts being fully heard and understood.
- The two TIC practices introduced a fundamental conceptual shift in the way the staff understood service users’ distress, by showing its links to trauma.
- As a result, staff felt increased compassion towards service users, and viewed distress and challenging behavior as threat responses rather than symptoms.
- The staff gained confidence in using new trauma-informed strategies to support service users more effectively and collaboratively, while minimizing the risk of re-traumatization.
- However, there remain systemic barriers to full integration of the new approach.

### 4.2 Themes: Summary of service user views

- Service users found the trauma-informed approach offered them new insights, knowledge and understanding about the origins of their distress.
- They found the stabilisation resources very helpful in managing their responses and reactions.
- Many of them were grateful for the opportunity to talk about painful past issues.
- Service users felt, in some cases in contrast to previous admissions, that they had been understood, supported and helped by the staff team during their stay on the ward.

For both staff and service users, and especially for the staff, the necessary first step seemed to be a ‘shift in ideology’ from ‘What’s wrong with you?’ to ‘What’s happened to you?’ (Sweeney et al., 2018). This transformation from a mainly medical to a trauma-informed paradigm seemed to facilitate all the other attitudinal and relational changes, along with the new conceptualization of service users’ reactions and responses not as ‘symptoms’ but as meaningful responses to situational or relational threats. The availability of stabilisation resources and new confidence in asking about, or disclosing, difficult emotional issues, helped to translate the new TIC model into practice.

### 4.3 Links to wider research

The findings support wider evidence that a psychosocial model of distress, as opposed to narrowly biological explanations, leads to increased feelings of compassion, and that this is an important foundation for enquiring and responding to experiences of trauma (Larkings and Brown, 2018; Lebowitz and Ahn, 2014; Toner et al., 2013). The present findings indicate the role of trauma-informed education and comprehensive team-based formulations, using models such as the PTMF, for creating compassionate teams. This finding is pertinent in the context of concerns about compassion fatigue within the NHS, and minimal pre-existing evidence on how to improve compassionate care within mental health settings (Crawford et al., 2013; Liberati et al., 2023). The responses from the service users, although more limited than those from the staff, support the hypothesis that TIC results in a more therapeutic experience, which equipped them with new skills and enabled them to process difficult life events that had contributed to their admissions. Although some service users have called for wider implementation of TIC (Sweeney et al., 2018) the authors were unable to identify any literature about the experience of TIC in adult acute mental health wards from the point of view of the inpatients. However, the core TIC values of safety, trustworthiness, choice, collaboration, and empowerment have been reported as helpful by trauma survivors (Diebold et al., 2021).

The findings indicate that TIC along with the PTMF may be especially beneficial in contexts where staff are frequently required to respond to high levels of distress. In such situations, the risk of counter-transference reactions in which staff act upon their own distress, may be high (Aremu et al., 2018; Chadwick and Billings, 2022). Staff described how, following team formulation, a more complete picture emerged of the service user, who was no longer viewed as someone who seeks to harm others, but as a someone who has been harmed themselves (Pickard, 2011). There is already some evidence for the helpful effects of team formulation (Division of Clinical Psychology, 2011), but the concepts of power imbalances and threat responses, which are central to the PTMF, were identified as especially valuable as an explanatory framework for distress and challenging behavior on the ward. The concept of power also helped staff to become more aware of the potential to harm patients through practices such as restraint and seclusion, thus inadvertently re-traumatizing them (Sweeney et al., 2016).

Consistently with other findings on the introduction of TIC, several staff participants described increased confidence in their ability to talk with service users about their past (Hall et al., 2016; Goldstein et al., 2018). Longitudinal research on services transitioning to TIC has demonstrated a reduction in the use of restrictive practices (e.g., Muskett, 2014). The present findings highlight the role of understanding specific behaviors differently, increased feelings of compassion towards service users, and having alternatives for responding to distress, in such trends. The findings add confidence to the existing evidence on the benefit of trauma-informed changes as part of meaningfully enhancing understanding and practice (Aremu et al., 2018; Azeem et al., 2011; Palfrey et al., 2019; Purtle, 2020; Williams and Smith, 2017).

As noted earlier, there appears to be only one other study that has explored changes in staff members’ understandings as a result of transition to trauma-informed care in an adult inpatient unit (Chandler, 2008). In this US clinic, similarly to the North London project, staff were trained in trauma-informed understandings and interventions, including educational material for the service users and the use of a ‘self-soothing’ cart with stabilisation resources. As with the North London project, significant reductions in restraint and seclusion were reported, and were attributed to the staff’s ‘changed perspectives’ (p. 366) and availability of alternative ways of managing distress. The report notes the need for ‘deep cultural change’ towards ‘new approaches to patient care (that) differ dramatically from their original training’ (p. 370).

In line with previous research, the main identified barriers to the integration of TIC on the North London wards were organizational, rather than patient-related (Chadwick and Billings, 2022; Huo et al., 2023). The demands of the ward environment were identified as monopolizing time and reducing staff availability for trauma-informed interventions. These barriers have also been identified elsewhere and support the view that it is difficult to successfully embed long-term change within systems struggling with staff capacity and sustained rises in demand (Evlat et al., 2021; Huo et al., 2023; Wilson et al., 2021).

### 4.4 Hypotheses of change

Nikopaschos et al. (2023) suggested a number of possible mechanisms of change for the reduced levels of restraint, self-harm and seclusion found in the quantitative evaluation of the TIC project (see ‘Evaluation aims’ above). In line with this, the qualitative interviews reported here support the suggestion that team formulations may have:

- increased staff understanding of service users from a trauma-informed perspective
- facilitated development of novel ways of managing distress on the ward
- increased staff understanding of the potential for re-traumatization by making explicit service users’ past experiences of trauma, and their meaningful links to current behavior
- increased staff understanding of the importance of creative alternatives to restrictive intervention
- strengthened relationships between staff and service users
- increased levels of empathy toward service users
- improved staff teamwork and communication.

As already noted, the PTMF’s emphasis on the role of power, and the conceptualization of ‘symptoms’ as threat responses, were also seen as helpful aspects of team formulation. The data suggest that the staff training in TIC and in the use and availability of stabilisation materials also contributed to these processes. The use of stabilisation strategies as alternatives to restraint and seclusion was described by a number of staff participants. No themes specifically related to improved staff teamwork and communication, although this may be inferred from the descriptions of a cohesive TIC approach across the team.

Nikopaschos et al. (2023) further suggested that the following factors might also be relevant in reducing levels of self-harm, restraint and seclusion:

- Reduced overall levels of service user distress.
- Service users feeling more able to cope with high levels of distress.
- Staff being able to respond to and de-escalate distress more effectively.

Overall levels of distress were not assessed as part of the project and therefore the first factor remains unproven. However, the data from service user participants provide many examples of service users’ increased confidence in managing their distress, while the staff participants consistently reported having more effective ways of de-escalating distress.

### 4.5 Limitations

As only seven of the 27 members of staff who attended the initial training program were judged to have attended enough sessions to have sufficient experience of the model, the results may not be representative of the larger group of staff. The same caveat applies to the eight service user participants, where an average of 316 service users are admitted to the unit each year (data taken from July 2017 and June 2022). Service users were required to have significant TIC input in order to participate, and therefore it is possible that others were not receiving TIC as intended. It is possible that both staff and service users with a more positive experience were more likely to respond, introducing a potential positive response bias. This skew toward more engaged or favorably oriented participants likely limits the generalizability of findings and may result in an overly optimistic representation of TIC’s impact. It is therefore important to interpret the results with caution, recognizing that experiences of disengaged or negatively impacted individuals remain underexplored. Future research should aim to include a broader range of perspectives to more fully capture the variability in experiences of TIC implementation.

The service user data was more limited, and the quality of this data may have been affected by a number of variables including:

- interviews being completed over the telephone to support ease of engagement but potentially negatively affecting rapport
- the specific sample of service users, currently engaging with secondary care mental health services with recent acute intervention, and the ongoing associated difficulties they may have been experiencing
- the limited scope of the trainee clinical psychology service-related research project, under which this data was collected.

It is also noted that formulations were not co-produced with service users during the initial Team Formulation phase. Several factors contributed to this, including one of the aims of Team Formulation to serve as an intervention for staff—supporting systems change and promoting trauma-informed thinking. In addition, the acute nature of service users’ presentations often limited their capacity to directly engage in structured formulation work during the initial phases of their admission. Clarke’s work on the Comprehend, Cope and Connect (CCC) model of psychologically-informed care offers an alternative and effective example of co-produced formulation in acute mental health settings. This model could be beneficial in informing future developments of the current approach (Araci and Clarke, 2017; Bullock et al., 2021; Clarke, 2021; Harris et al., 2023; Riches et al., 2024).

Finally, given the small sample size and exploratory nature of the study, data saturation was not achieved. This limits the depth and breadth of themes captured and should be considered when interpreting the findings.

### 4.6 Clinical implications

Together, the implementation of the TIC training program and use of PTMF Team Formulation sessions have facilitated a structured change process for transitioning a ward environment to a trauma-informed model. The results represent a significant achievement in a setting previously dominated by narrowly diagnostic understandings of distress, with limited access to psychosocial interventions to support recovery, and facing all the usual challenges of high demand and under-resourcing.

The costs of the project were minimal, since most of the training was carried out in-house. However, as several staff participants noted, structural change is needed in order to realize the full benefits of the approach within a ward environment dominated by other pressures.

The model was compatible with the practice of the professional groups participating in the study, including Occupational Therapists, Nursing and support staff. The changes correspond to several aims of The Long-Term Plan (NHS, 2019), including the need for improvements in crisis care, increased access to therapeutic interventions and the delivery of TIC by a psychologically-informed workforce.

The service program was included as an exemplar practice in recent guidance by NHS England, which outlined a directive and future vision for England’s inpatient mental health care (NHS England, 2023). The project has subsequently been adopted by other NHS services, in consultation with two of the PTMF authors. The philosophy underpinning both TIC and the PTMF is also in keeping with the direction of travel indicated in a recent WHO/UN report: ‘Mental health and well-being are strongly associated with social, economic, and physical environments, as well as poverty, violence, and discrimination. However, most mental health systems focus on diagnosis, medication, and symptom reduction, neglecting the social determinants that affect people’s mental health … There is an overreliance on biomedical approaches to treatment options, inpatient services and care, and little attention given to social determinants and community-based, person-centered interventions’ (WHO/UN, 2023).

It is worth noting that TIC can be translated into practice in a number of different ways and can be delivered in narrow or insensitive versions (Sweeney and Taggart, 2018). TIC needs to be supportive of individual needs and preferences, and the data gives examples of some situations in which this may not have been the case (e.g., one participant felt that there was too much emphasis on discussing the past). In addition, not every psychiatric inpatient has a trauma history as traditionally understood, although the general principles of TIC (safety, trustworthiness, choice, collaboration and empowerment; Fallot and Harris, 2006) should apply across all aspects of a trauma-informed system.

As Chandler (2008) noted, TIC if properly understood, represents a paradigm that is not just different from, but antithetical to, the currently dominant one; both the ideas and the practices ‘differ dramatically from (the staff’s) original training’ (p. 370). As they also note, and as these evaluations demonstrate, ‘Cultural change takes both individual commitment and structural supports’ (p. 370). Despite these challenges, the project illustrates the highly positive changes that can be achieved with commitment and perseverance.

### 4.7 Future research

Further research into staff and service user inpatient experiences of TIC and the role of the PTMF in supporting it, is needed to validate and build on these findings. As noted above, there is very little research on service user experience of receiving TIC in mental health settings, and this is a gap that needs to be filled.

A number of additional measures would help to throw light on the findings of both this and the previous paper. For example, we do not currently have data on whether there was an impact on length of stay, re-admission rates, use of medication, likelihood of recovery, and overall costs. Follow-up and further data collection would help to fill this gap. It would also be useful to explore in more detail the ways in which the PTMF can support service users to construct or co-construct new narratives, in addition to learning new skills.

## 5 Conclusion

The findings indicate that Trauma-Informed Care drawing on PTMF principles can play an important role in bringing about culture change on adult acute inpatient wards, by offering a new way of conceptualizing service users’ difficulties. This seems to form the foundation of a change process in which staff are able to contextualize service user behaviors and reactions, which in turn enables them to respond more compassionately and effectively. As a result, rates of self-harm, seclusion and restraint are reduced (Nikopaschos et al., 2023). Service users similarly report that they have been introduced to valuable new information and skills; that staff were supportive; and that the stay of the ward admission was beneficial, equipping them with new strategies to manage their distress. However, systemic barriers to more widespread implementation of this approach still remain.

## Data Availability

The raw data supporting the conclusions of this article will be made available by the authors, without undue reservation.
