# Supplementary material for: Trauma-Informed Care on mental health wards: staff and service user perspectives
Source: Front Psychol. 2025 Sep 19;16:1578821. doi: 10.3389/fpsyg.2025.1578821 (PMC12494177; doi:10.3389/fpsyg.2025.1578821)
Supplement: Supplementary file 1 [file Data_Sheet_1.docx]

**APPENDIX A - The Power Threat Meaning Framework Core Questions**

1. What has happened to you? (How is power operating in your life?)
2. How did it affect you? (What kind of threats did this pose?)
3. What sense did you make of it? (What is the meaning of these situations and experiences to you?)
4. What did you have to do to survive? (What kind of threat responses are you using?)
5. What are your strengths? (What access to power resources do you have?)
6. What is your story? (How does this fit together?)
