# Supplementary material for: Trauma-Informed Care on mental health wards: staff and service user perspectives
Source: Front Psychol. 2025 Sep 19;16:1578821. doi: 10.3389/fpsyg.2025.1578821 (PMC12494177; doi:10.3389/fpsyg.2025.1578821)
Supplement: Supplementary file 3 [file Data_Sheet_3.docx]

**APPENDIX C - Interview schedule for staff**

1. How have the formulation meetings and training sessions impacted your understanding of mental health difficulties?
2. How have the formulation meetings and training sessions impacted your understanding of the influence of trauma and adversity on mental health?
   Prompt: For example, experiences of different forms of abuse, bullying, neglect, marginalisation, discrimination.
3. How does this compare to your understanding of the causes of mental distress previously?
4. Are there any other ways the formulation meetings and training sessions have impacted your understanding and knowledge?
5. How have the formulation meetings and training sessions impacted your engagement with service users on the wards?
6. Are there any other ways the formulation meetings and training sessions have impacted your practice on the wards?
7. How does this compare to your engagement and practice previously?
8. What are the challenges or barriers to trauma-informed practice on the ward?
