# Supplementary material for: Trauma-Informed Care on mental health wards: staff and service user perspectives
Source: Front Psychol. 2025 Sep 19;16:1578821. doi: 10.3389/fpsyg.2025.1578821 (PMC12494177; doi:10.3389/fpsyg.2025.1578821)
Supplement: Supplementary file 4 [file Data_Sheet_4.docx]

**Appendix D - Interview schedule for service users**

1. Have you had previous hospital admissions? (If no, go to question 4). If yes: How did this admission compare to previous admissions?
2. Did you receive a copy of the manual? If yes, did you find it a helpful resource and why?

Prompt: Do you still have access to the manual following your discharge?

1. Have you used the stabilisation interventions following your discharge from the ward? (If no, go to question 8)
2. Could you rate on a scale of 1-5 how helpful you have found the stabilisation interventions in managing distress since your discharge? (Rating scale described: 1: very unhelpful. 2: unhelpful. 3: no opinion / neither helpful or unhelpful. 4: helpful. 5: very helpful)
3. Could you tell me a bit more about why you rated the stabilisation interventions as [insert rating number]?

Prompt: Any specific skills/interventions/experiences you found helpful/unhelpful? Prompt: Have you noticed any differences in using the interventions since discharge compared to when on the ward?

1. Have there been any barriers to engaging with the stabilisation interventions since your discharge?

*‘At the beginning of our call, I asked you some questions about the stabilisation interventions on the ward. Stabilisation is one of the important phases within trauma-informed approaches. Information on trauma-informed approaches was included in the first booklet of the manual. Trauma-informed approaches acknowledge the relationship between people’s life experiences and the development of mental health difficulties. We know that it is very common for people who use mental health services to have been through difficult life events.’*

1. Aside from stabilisation interventions, do you think that trauma-informed approaches were integrated into your recent admission? (i.e. do you think that your history/previous experiences were taken into account?).

Prompt if yes: In what ways/examples. What was your experience of this?

Prompt if no: Can you tell me more? What was your experience?
