# Supplementary material for: Trauma-Informed Care on mental health wards: staff and service user perspectives
Source: Front Psychol. 2025 Sep 19;16:1578821. doi: 10.3389/fpsyg.2025.1578821 (PMC12494177; doi:10.3389/fpsyg.2025.1578821)
Supplement: Supplementary file 5 [file Data_Sheet_5.docx]

**APPENDIX E - Staff Interviews: Example of codes with data extracts**

| **Code example** | **Participant number and data extract** |
| --- | --- |
| Awareness of potential for re-traumatisation | P2: Someone who has been abused … they feel powerless ... when they come to the ward, we’re using that same power.  P3: It helps us understand why they’re acting that way as well … what are their responses to these power imbalances ... like when they’re sectioned or if they’re not feeling safe … in terms of them being detained and stuff like that, and loss of freedom.  P4: I think sometimes as well people will stop behaving in a certain way if they're not scared of you laying hands on them.  P4: You’re just sort of reinforcing abuse that happens to them. |
| Behaviour as a threat response | P1: If they self-harm or if they isolate themselves ... it’s how they respond to the trauma.  P3: It helps us understand why they’re acting that way as well ... what are their responses to these power imbalances ... like when they’re sectioned or if they’re not feeling safe.  P4: You start to think oh yeah actually that makes sense and that fits as a threat response or that’s how they interpret things.  P4: Things we would normally look at as symptoms, you look at it as like a threat response.  P5: Someone’s been through sexual abuse and then afterwards they start taking drugs just to cope.  P6: Understanding where maybe this aggression or intimidation comes from ... maybe from a place of fear so, through trauma. |
| Contextualising ‘symptoms’ | P2: Knowing someone’s background ... it helps you understand the reason why they’re doing it ... why the symptom being there.  P3: [the framework] gives you so much more background to things that you would look at as symptoms ... when you actually look at what they responded to ... then tie it into all their experiences throughout their life.  P4: [before attending] I thought everybody exhibited the same kind of symptoms ... so like someone with a personality disorder would be more, like unpredictable ... I just generalised everybody in the same sort of way.  P4: You only get information [in handover] on their presentation and it is sort of dehumanising in a way to be like, this is their behaviour ... not in the past this has happened.  P6: You might be able to understand the reason that this person is behaving like this, because of the trauma they’ve experienced in the past.  P7: A deeper understanding, rather than just looking at them, you know, as they’re presenting, rather than not having their background. |
| De-escalation | P1: So every time he was getting like that ... anxious ... and high, it was reminding him like remember your self-soothing items.  P2: Instead of just going straight for the medication, because if you put them in restraints, you’re making the situation worse.  P2: The patient is kicking off, and you tell them...mindfulness - ‘bring your mind back to the present' ... things like that.  P3: When you're being abused by somebody verbally sometimes that can grate off on you, but then you have that constant reminder to tell you that "Oh they don't ... mean it'. P4: If I know they've done grounding techniques, or distraction and distancing, then I can encourage them to look at them when they're really distressed, it might completely reverse that. P4: You feel so much more compassionate about those incidences.  P6: Not taking things too personally, understanding where maybe this aggression or intimidation comes from. |
