# Supplementary material for: Trauma-Informed Care on mental health wards: staff and service user perspectives
Source: Front Psychol. 2025 Sep 19;16:1578821. doi: 10.3389/fpsyg.2025.1578821 (PMC12494177; doi:10.3389/fpsyg.2025.1578821)
Supplement: Supplementary file 6 [file Data_Sheet_6.docx]

**APPENDIX F - Initial themes and sub-themes, and extracts from the service user interviews**

| **Theme** | **Sub-theme** | **Participant number & data extract** |
| --- | --- | --- |
| Benefits of psychology | Provides something additional | P1: The stabilisation interventions kept me going in hospital.  P1: I was enjoying the groups, I was able to participate and talk about the issues and subjects that were covered. The manual gave me something to work towards, I was able to achieve something.  P1: I had my psychologist she was very helpful … the assistants were great.  P3: Very calming, made you think about things.  P4: Very helpful because it’s not something that I knew.  P4: [comparing to previous admission] They didn’t go through skills. They did not talk about stabilisation like on this ward. This ward very helpful. It made things easier to accept for me.  P6: It helped me understand what I was going through and how to manage it in case it comes back. It was relaxing, a way to help get through the day.  P7: I felt like an individual not a tick on a sheet.  P8: Very helpful, they were really good for me.  P8: It’s the only thing I brought out the hospital with me, how to manage myself, it’s been the main thing for me. |
|  | Coping skills | P1: Mindfulness helps you to clear your head.  P3: Meditation and mindfulness skills were very helpful. I would get into a bit of a rage, get upset, then use the skill and it would calm me down.  P3: [self-compassion skill] Had never heard of the term before. I don’t like myself very much, but we are who we are, and helped me to deal with it.  P4: The skills, they teach you how to manage stress, how to put it across to the doctors. I found that very helpful.  P4: The effective communication skill was really helpful.  P5: It was helpful…mindfulness.  P7: The meditation was helpful, trying to be in the moment rather than thinking about everything that has gone on that you can’t change…instead of worrying about stuff that you can’t control, so you know just try and be in the moment.  P8: Very helpful, they were really good for me…everything that I got…was really helpful. |
| Use of psychology | Ward environment | P7: The meditation/mindfulness, taking myself away from when things got too noisy, take myself away into my own room, put the radio on.  P8: I used them mainly when I had the sessions and in the evening time mainly for me, I would use the techniques that I learnt then to keep me calm. |
|  | Long-term use | P3: I still do the meditation at night when I can’t sleep.  P8: I still use all the breathing techniques at the moment.  P8: When I’m home and I start thinking about things, I go back to my managing distress in the moment plan and it takes my mind off it.  P8: I’ve read a lot more [manual] now that I’ve left hospital, trying out new techniques, putting things in place. |
|  | Preference for 1:1 | P3: 1:1 is easier. In the group, it would be a bit much, more people around. When 1:1, easier to focus on things … more comfortable.  P4: Assistant psychology was very helpful, the one to one sessions. She talked through how to … when you’re feeling anxious, how to understand yourself and how to put what you are trying to say across to other people.  P6: I mainly went to 1:1s rather than the group, found the 1:1 helpful.  P7: I prefer the 1:1, that is more because … I’m socially awkward … I prefer the 1:1 because there is no hiding, it’s just you.  P8: I felt the 1:1 was probably better, I did find the groups helpful but don’t like groups as much. |
| Barriers | Harder on your own | P1: I was meant to do the rest of the skills and never got round to it. I just stopped doing them … It is hard to engage with the skills on your own … I haven’t thought about the stabilisation booklet much.  P7: When you’re on the ward, it’s there, you know the groups are coming up, the 1:1 sessions are in the afternoon, there is the reminder that its going on, so you engage more in the hospital  P7: Not in the forefront of my mind, it’s not like I don’t want to be doing it because it doesn’t help, I just can’t remember it. |
|  | ‘Life’ gets in the way | P1: It is easier to engage with in hospital … when you’re at home, you think, oh I’ll do it in a minute, and so on, but don’t get around to doing it. When you’re at home, and you’ve got problems, you don’t think about doing the stabilisation skills. Cause you have problems on your mind.  P3: Lots of things going on, I’ve been extremely busy.  P5: Just finding the time to do them.  P6: In the outside world, there is so much more going on and much more people  P7: It’s now been put to one side, and you start to do other things, getting back into a routine and forget it. |
|  | Feeling ready | P2: My mental state is so poor, I find it hard to take it in…I decided to switch off…if I’d been a bit more well, then brain engage more.  P2: Skills would have been helpful for me…when not that bad…  P3: [talking about previous admission] Previous one I did not bother to engage. There was no staff around to talk to. This time I engaged and spoke to staff … one day I thought sod it and decided to try and engage. I’m happy I did as it helped me. |
| Opportunity to talk | Talking and mental health | P1: [talking about previous admission] It wasn’t really about talking, more about keeping busy. It’s a positive difference, the fact that I was able to talk during my admission. Talking helps.  P1: I was overwhelmed … I would cry and I needed to cry … I was bottling it all up. That’s why I was slow and had my illness, because I don’t talk.  P3: I kept it in … hospital and talking helped me to come through the other side.  P4: Talking to friends and family when you are upset about something, instead of trying to figure out yourself.  P7: If you needed someone to speak to they are there you’ve only got to ask … maybe that’s what they need, someone to break the ice.  P7: You can either go with it, or ignore it, push it away and if you push it away you aren’t helping yourself … you need to learn to manage it and face up to the reason why you’re there.  P8: It was a good decision, I needed to open up to someone otherwise it would have made me worse.  P8: It made me feel a lot better because I’ve never really opened up to people, always bottled everything up. |
|  | Connecting with other service users | P1: I like to hear other people’s opinions.  P1: I was completing the manual in the communal area of the ward. I had people to talk to, and I could ask their opinions.  P1: I was able to participate and talk about the issues and subjects that were covered. I had great conversations.  P6: Was also a way to be able to interact with the other patients. I have also spoken to other people about the skills and shown them, other patients from the ward, and they also say they find it helpful. |
|  | The past | P1: We did my timeline, linking past to my present, it really helped.  P1: I didn’t realise of events that happened in my life until I did the timeline.  P3: My history was taken into account by the team … staff asked me about it [the past], asked the right questions.  P4: It’s okay for the past to be taken into account. But keep on bringing up, numerous times. That is dragging person back in that mood or mind space when they are depressed.  P4: In classes we were taught to forget the past and move on. When I said that to the doctors, we have to ask about the past … the repeatedness of the questions felt unhelpful, retelling story time and time again … very negative.  P5: The doctors asked me but I don’t want to think about the past, and wanting to focus more positively about the future. I think about the future not the past. You sometimes need the past, the past story will teach you. |
